# Functionalized Fullerenes and Their Applications in Electrochemistry, Solar Cells, and Nanoelectronics

**DOI:** 10.3390/ma16031276

**Published:** 2023-02-02

**Authors:** Maksim Paukov, Christian Kramberger, Ilia Begichev, Marianna Kharlamova, Maria Burdanova

**Affiliations:** 1 Center for Photonics and 2D Materials, Moscow Institute of Physics and Technology, 9 Institutsky Lane, 141700 Dolgoprudny, Russia; 2 Faculty of Physics, University of Vienna, Strudlhofgasse 4, 1090 Vienna, Austria; 3 Center for Photonics and Quantum Materials, Skolkovo Institute of Science and Technology, 143026 Moscow, Russia; 4 Centre for Advanced Material Application (CEMEA), Slovak Academy of Sciences, Dúbravská cesta 5807/9, 854 11 Bratislava, Slovakia; 5 Institute of Materials Chemistry, Vienna University of Technology, Getreidemarkt 9-BC-2, 1060 Vienna, Austria; 6 Laboratory of Nanobiotechnologies, Moscow Institute of Physics and Technology, Institutskii Pereulok 9, 141700 Dolgoprudny, Russia; 7 Institute of Solid State Physics, Russian Academy of Sciences, 142432 Chernogolovka, Russia

**Keywords:** fullerene, optoelectronic, electrochemistry, solar cell, diode, nanoelectronics

## Abstract

Carbon-based nanomaterials have rapidly advanced over the last few decades. Fullerenes, carbon nanotubes, graphene and its derivatives, graphene oxide, nanodiamonds, and carbon-based quantum dots have been developed and intensively studied. Among them, fullerenes have attracted increasing research attention due to their unique chemical and physical properties, which have great potential in a wide range of applications. In this article, we offer a comprehensive review of recent progress in the synthesis and the chemical and physical properties of fullerenes and related composites. The review begins with the introduction of various methods for the synthesis of functionalized fullerenes. A discussion then follows on their chemical and physical properties. Thereafter, various intriguing applications, such as using carbon nanotubes as nanoreactors for fullerene chemical reactions, are highlighted. Finally, this review concludes with a summary of future research, major challenges to be met, and possible solutions.

## 1. Introduction

Studies on nanomaterials have recently increased worldwide due to the novel properties associated with a typical large-aspect ratio. This offers the possibility for the exploration of potential applications in nanoscale devices. Nanostructures can be categorized into zero-dimensional (0D), one-dimensional (1D), and two-dimensional (2D) nanostructures. Among them, 0D nanostructures have all their dimensions on the nanometer scale, while the rest have one and two dimensions on the nanoscale. These include quantum dots, nanoparticles, and fullerenes. Any nanomaterial featuring atoms in the shape of a hollow sphere is called fullerene.

One of the most important 0D materials, fullerene has attracted considerable attention in different fields of science since its discovery in 1985. Kroto and coworkers synthesized a carbon species containing 60 atoms that formed spontaneously in relatively high abundance [1]. C_60_ and also C_70_ were exceptionally stable, in comparison with smaller metastable species, such as C_28_, C_36_, and C_50_. The field of fullerene chemistry expanded as these fullerenes became available in workable amounts.

Fullerenes have many remarkable properties, stemming from the topological fact that these molecules are borderless, uncharged, and, in the absence of boundaries, have no unpaired electrons [2]. Fullerenes are excellent conductors of heat and electricity, and they possess exceptional tensile strength [3]. The C_60_ molecule undergoes a wide range of novel chemical reactions. It readily accepts and donates electrons, a behavior that suggests possible applications in batteries and advanced electronic devices. Such properties result in the usage of fullerenes in optoelectronics, medicine, and other areas. Practical applications, however, are limited since pure fullerenes are insoluble in water. 

Fullerenes, with their low dimensionality, quantum confinement, and shape effect, have properties that bulk materials do not possess. Intensive research has focused on the functionalization of fullerenes and their morphology, along with their chemical and physical properties [4]. The number of articles that have been published on the topic is increasing exponentially. Firstly, biomedical applications require the dispersion of fullerene in a solvent, with aqueous dispersions being preferred because of biocompatibility, safety, or environmental concerns. Fullerenes’ solubility in solvents expands the options for processing it in solutions, which opens up the opportunity to form the sort of uniform films that are required for coatings, electrodes, etc. Finally, a deep understanding of the interaction of fullerenes with solvents requires improved purification via cheaper and more scalable methods. Understanding this solubility is also important for sorting techniques. Functionalized fullerenes have been used widely, with potential applications in solar cells, drug delivery systems, and materials science in general [5,6,7]. In our article, we would like to offer a comprehensive review of previous research activities focused on the functionalization of fullerenes. 

## 2. The Methods of Synthesis of Fullerenes 

The first possibility of creating a hollow spherical carbon molecule was suggested in 1966 by David Jones [1]. He wrote that “it would be a spherical shell of a sheet-polymer like graphite, whose basic molecule is a flat sheet of carbon atoms bonded hexagonally rather like chicken wire”. This suggestion was forgotten for several years until Osawa theoretically predicted the possibility of polyhedral carbon clusters in 1970 [2]. He predicted the C_60_ molecule, which is made up of 60 carbon atoms in the structure of a truncated icosahedron. For a long time, no one could either confirm or refute these predictions experimentally. In 1985, Kroto et al. studied the mass spectra of graphite vapor obtained by the laser irradiation of a solid sample and found lines that they attributed to C_60_ and C_70_ molecules. 

At the beginning of the 1990s, many scientific articles were published about new methods for producing fullerenes and the influence of various parameters on their formation. In general, for the synthesis of fullerenes, temperatures of 1200 °C or higher are necessary to bring the carbon source into the gas phase and efficiently form molecules. Therefore, a number of methods, such as electric arc, plasma torch, shock waves, high-temperature treatment, electron irradiation, etc., were proposed to achieve the high temperature necessary. The strain energy of C_60_ is huge and the heat of combustion tests place the value somewhere in excess of 600 kcal/mol. 

The most common method of fullerene synthesis is via laser ablation. Smalley and co-workers [3] were the first to demonstrate the viability of this technique. Briefly, the team used a long quartz tube mounted inside a temperature-controlled tube furnace. This quartz tube was attached on the front end to an aluminum inlet block that allowed the researcher to control the gas flow and pressure. The rear end was usually connected to a vacuum pump. Noble gas at several hundred Torr and temperatures of 1200 °C were used for fullerene synthesis. Smalley et al. detected the signal of LaC_60_ in the mass spectrum, which indicated the presence of endohedral LaCl_3_. They used the symbol “@” for identifying the encapsulation of metals in fullerenes. The laser evaporation of the mixture of graphite and BN resulted in the formation of fullerenes and boron-containing analogs, such as C_59_B and C_54_B [4].

**Figure 1:**
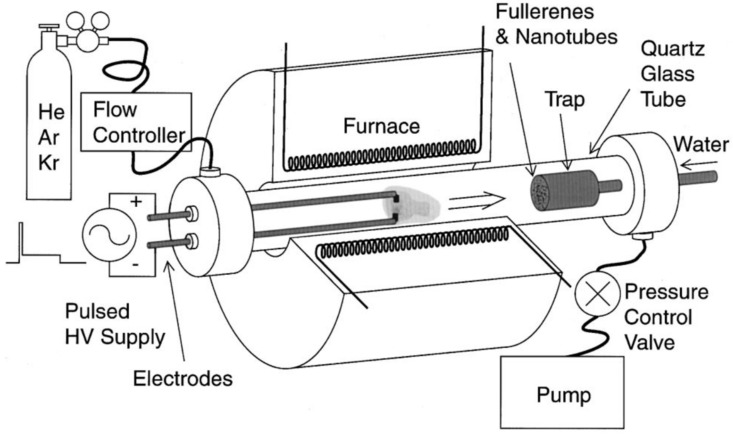
Schematic of a high-temperature pulsed arc discharge setup. Reprinted with permission from Ref. [12]. Copyright 2000, American Chemical Society.

The synthesis of fullerenes via the arc discharge method was accomplished by Krätschmer and Huffman et al., who introduced the direct-current technique [5,6]. The arc discharge method is one of the ways to produce gram-sized quantities of fullerenes [7,8]. In the arc discharge method, resistive graphite electrodes were heated to a temperature of about 2 × 10^3^ °C in an inert atmosphere of 100–200 Torr, with either helium or argon gas (Figure 1). This process involves vaporizing the carbon from graphite into a high-density gas flow, using a focused pulsed laser. The addition of transition metals or metal oxide catalysts has also been applied for improving the yield of fullerenes [9,10,11,12]. In [13] the authors introduced N_2_ gas into the arc discharge furnace, which occasionally led to the formation of Sc_3_N@C_80_. Later, a number of works using the same method introduced NH_3_, SO_2_, O_2,_ and numerous organic and inorganic molecules [14,15,16,17]. Some of them resulted in the formation of filled fullerenes, such as ErSc_2_N@C_80_, Sc_3_N@C_84_, Sc_3_C_2_@C_80_, Sc_4_C_2_@C_80_, Sc_4_O_2_@C_80_, YCN@C_82_, H_2_@C_60_, Er_x_Sc_3–x_N@C_80_ (x = 1–3), and TiM_2_N@C_80_ (M = Sc, Y) [9,10,11,12,13,14,15,16,17,18]. Multi-shell fullerenes have a cage-in-a-cage concentric structure, such as the double-shell C_60_@C_240_ or triple-shell C_60_@C_240_@C_560_, and were synthesized using the arc discharge technique [19]. The metal-free growth of fullerenes is still under debate.

**Figure 2:**
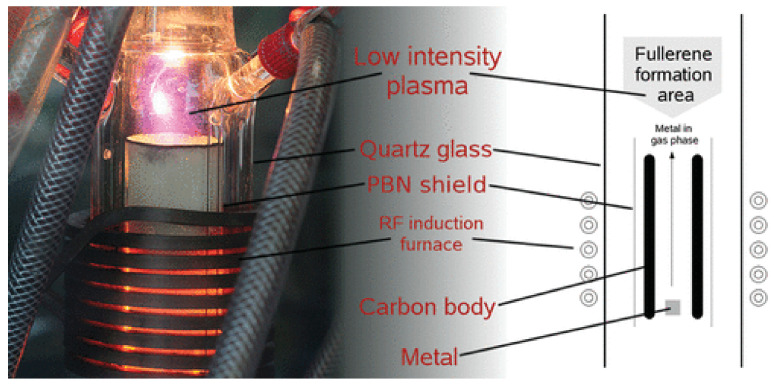
A photograph and model of the production chamber in the RF furnace. Reprinted with permission from [28]. Copyright 2010, American Chemical Society.

The radio frequency (RF) plasma furnace method can be utilized to produce empty fullerenes in very high yields [20]. It is based on the evaporation of metal ions and carbon in the fullerene formation zone in radio frequency-powered plasma; nitrogen radio-frequency plasma leads to the formation of nitrogen (N@C_60_) (Figure 2) [21]. The radio-frequency plasma method has been widely adopted for the synthesis of numerous types of endohedrally functionalized fullerenes. Among them, La@C_82_, M@C_2n_ (2n < 80), CCF-Sc_4_C_2_@C_80_, NCF-Sc_3_N@C_2n_, N@C_80_, and so on were synthesized [22,23,24]. Recently, fullerene-bonded-CNTs were successfully synthesized using radio-frequency plasma (RF plasma) treatment [25]. 

Many different methods for the chemical synthesis of fullerenes have been envisaged. Assembling two identical hemispherical hydrocarbons was proposed as a promising route for the synthesis of fullerenes. The introduction and stabilization of curvatures or pyramidalizations in the carbon network is the primary problem in fullerene production using chemical routes. The first synthesis of the corannulene molecule showed the possibility of adding curvatures in anaromatic molecules [26,27]. Many other fragments of fullerenes were studied and it was proposed that fragments with an electronic character that was more similar to that of C_60_ should favor the formation of C_60_ [28]. It was shown that three main fragments, comprising decacyclene (C_36_H_18_), tribenzodecacyclene(C_48_H_24_), and trinaphtodecacyclene (C_60_H_30_), react via pyrolysis to form C_60_ fullerenes [29]. The first rethional synthesis of C_60_, proposed by Scott and co-workers, suggested the pyrolytic synthesis of C_60_ from the C_60_H_27_Cl_3_ molecule [28].

**Figure 3:**
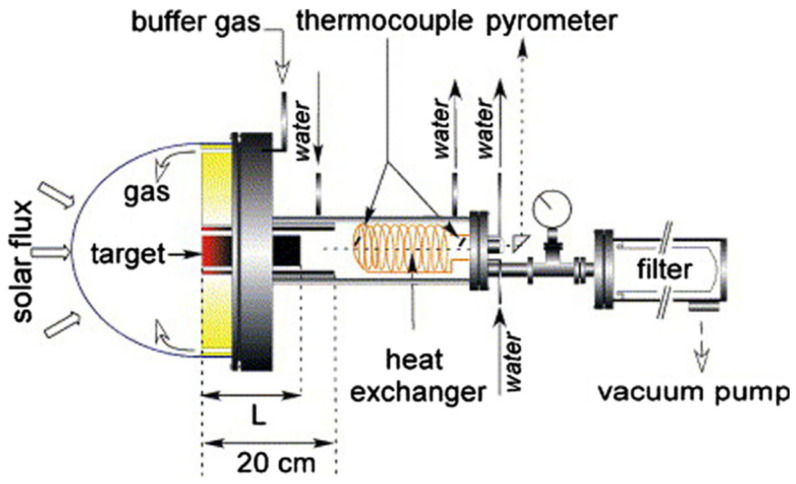
Typical scheme of the setup used in solar flux experiments. Reprinted with permission from Elsevier (Copyright 2004)) [30].

Fullerenes have also been produced by the direct vaporization of carbon in focused sunlight [30]. Large-scale solar furnaces are uniquely capable of mass-producing fullerenes from green energy (Figure 3). Methods based on graphite vaporization and burning hydrocarbons in a fuel-rich flame have been widely investigated [31,32]. Research has determined the yields of fullerenes, over a range of conditions, in low-pressure premixed laminar flames of different mixed combustible materials. In particular, combusting a mixture of benzene and oxygen at a ratio of 1:1 in a laminar flow resulted in a high yield of fullerene species. Great progress was made in the synthesis of fullerenes and also in the understanding of their formation process. The hydrocarbon combustion approach for fullerene synthesis is the most effective of all these procedures. This technique is capable of producing multiple tons of fullerenes per year. However, the actual mechanism of fullerene creation in flames is as yet unknown.

The above-mentioned methods and theories have been developed to understand the formation of fullerenes. According to the general theory, the carbon source is first reduced to its smallest components, such as carbon atoms and, probably, carbon dimers, which undergo a series of processes before recombining to form fullerenes, within a specific pressure and temperature range [3]. However, this process was not confirmed later by experimental observations. The icospiral particle nucleation scheme was also proposed to explain how fullerenes are formed [33]. According to this model, the icospiral nucleation process begins with a C_20_ molecule that resembles corannulene and has a pentagon encircled by five hexagons in its structure. This highly reactive structure expands and has a tendency to produce open spiral shells by accumulating microscopic carbon particles that are cleaved upon adsorption onto the surface of such shells. The subsequent creation of fullerenes is explained by the sporadic statistical closure of a network with the proper arrangement of pentagons [33]. The annealing of carbon clusters is another method for producing fullerenes [34]. These carbon clusters could be in bi- or tri-cycles, with between 34 and 60 atoms, chains, or rings that stick together and subsequently anneal [35].

Later on, it was suggested that fullerenes originate from a nanoscale intermediate phase (or hidden phase) [36]. Non-equilibrium circumstances lead to the formation of clusters of this intermediate phase, which was named kvataron. Based on the results of quantum chemical molecular dynamics simulations of the dynamics of self-assembling in a hot carbon vapor that is far from thermodynamic equilibrium, Irle and colleagues proposed a mechanism called “the shrinking hot giant road to fullerene formation”, in which fullerenes are formed by the shrinking of giant fullerenes [37]. 

**Figure 4:**
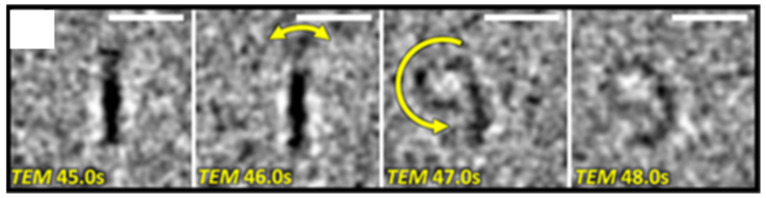
SMART-EM exploration of the molecule-to-molecule transformation of polycyclic aromatic hydrocarbon to C_60_ on graphene. Yellow arrows indicate the direction of motion [39]. Copyright 2021, American Chemical Society.

Aberration-corrected transmission electron microscopy directly visualized fullerene synthesis from a graphene sheet and a Truxene derivative, C_60_H_30_ (Figure 4) [38,39]. Four critical steps of fullerene formation were proposed, using quantum chemical modeling: (i) the loss of carbon atoms at the edge of graphene; (ii) the formation of pentagons; (iii) the curving of graphene into a bowl-shaped structure; (iv) the closing fullerene structure.

The main issue in fullerene synthesis is the extraction of fullerenes from the crude carbon soot obtained during the synthesis process. In addition, different types of fullerenes (C_60_, C_70_, etc.) and carbon allotropes are mixed together in the synthesized material. The most typical method for the extraction of fullerenes is solubilization in different solvents. Among them, pyridine, heptane, hexane, benzene, and toluene have been widely used. The solid carbon soot can be placed in a Soxhlet separator in the presence of these solvents. As with many nanomaterials, the extracted fractions become colorful, which confirms the sorting protocols. The Soxhlet separator usually results in the extraction of C_60_ and C_70_ fullerenes. Further sorting can be performed analogously to the CNT methods [40]. In another study, an initial attempt was made to separate the most prevalent fullerenes, C_60_ and C_70_, from Soxhlet extracts of carbon soot using traditional open-column chromatography [41]. Different stationary phases were used, such as phenylglycine, dinitroanilinopropyl silica, polymer, and so on [42]. Vacuum sublimation is another important method that allowed researchers to achieve the high-purity extraction of fullerenes. The evaporation of carbon soot in a vacuum or inert gaseous phases resulted in the evaporation of fullerenes and their consequent collection in the air-cooled chambers. 

Among other methods, laser ablation yielded stable fullerene clusters. However, this method requires a laser with high intensity, which is expensive. In addition, extremely pure graphene disks are required and give a relatively low yield. These two points make this method less desirable for commercial production. The arc-discharge method is known as a high-yield technique, which can be used in mass production despite the harsh conditions required. Meanwhile, synthesis methods are expensive due to the reactant used and require the precise following of protocols, although a relatively high yield was achieved. 

## 3. The Methods of Functionalization of Fullerenes 

Fullerene chemistry has become very widely studied and well-understood. A large number of functionalization routes have been proposed. In particular, some of them are similar to SWCNT; details can be found in our previous article [43]. 

**Figure 5:**
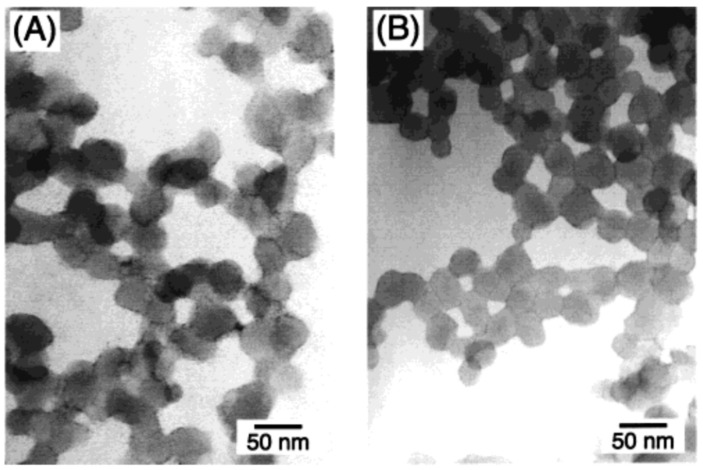
TEM image showing the C60 clusters (**A**) and C70 clusters (**B**) in an aggregated state [50].

Fullerenes have pure solubility in most commonly used solvents and are present in their aggregated form (Figure 5) [44]. The best solubility performance of C_60_ was observed in toluene and hexane. However, in most cases, it is necessary to apply high pressure and high temperatures [45,46,47]. In a previous study [47], the solubility of fullerene C_60_ in toluene was measured at various temperatures, ranging between 278.2 and 308.2 K, and pressures of up to 340 MPa. In addition, fullerene solutions were investigated at temperatures between 258.2 and 298.2 K under atmospheric pressure. A solubility maximum of C_60_ in toluene and carbon sulfide was observed around 0 °C, while the solubility in o-xylene is best at around 30 °C [45]. Further improvements in solubility can be achieved by covalent and non-covalent functionalization. 

Several stable dispersions of fullerenes in polymer composites have been reported using covalent interactions. In particular, composite fullerene-polymer nanofibers were synthesized using copolymers, such as poly(3-hexylthiophene) and phenyl-C_61_-butyric acid methyl ester. Importantly, this improved their performance in organic photovoltaic devices [48]. In addition, polymer-based fullerene nanocomposites showed different results in comparison to SWCNT, which is dispersed in the same polymers [49]. Fullerenes functionalized with anti-aromatic isophlorin molecules also showed great stability in both the liquid and gaseous phases. This research demonstrated that anti-aromatic π surfaces are as good as aromatic surfaces for binding fullerenes [50]. 

Fullerenes can be dispersed in an aqueous medium in micelle form, using various surfactants. Tween 20, Tween 60, Tween 80, Triton X-100, PVP, polyoxyethylene (10) lauryl ether, n-dodecyl trimethylammonium chloride, myristyl trimethylammonium bromide, and sodium dodecyl sulfate have been widely used in numerous applications [51]. Among them, lipid peroxidation tests showed that the micellar solutions of fullerene with Triton and polyoxyethylene lauryl ether maintained high radical scavenging activity. 

Fullerenes can be also functionalized via covalent interactions with molecules. They were actively oxidized in different atmospheres, such as ozone, oxone monopersulfate, oxygen, etc. The oxidation of fullerenes and their derivatives is hard to achieve, due to the high energy barriers. Ozone is an important oxidant as it is present in many gas and organic species. It was shown that the degradation of fullerenes occurs when they are stored under light illumination in oxygen. The consequent opening and fragmentation of the fullerenes were observed under ambient conditions [52]. Oxidation reactions using O_2_ and H_2_O_2_ have resulted in improved water-solubilization [53]. Recently, Staudenmaier’s method was used to produce both epoxy and hydroxy groups on the surface of fullerenes and thereby improve the solubility of fullerene in polar solvents [54]. 

Fullerene hydrogenation is found to offer a promising route for reversible hydrogen storage. Hydrogenated fullerenes, called “fulleranes”, were produced in a Birch−Hückel reaction, resulting in C_60_H_18_−C_60_H_36_ as the main products. Higher levels of hydrogenation were achieved under high pressure [55,56]. In addition, the reduction of fullerenes with Zn and concentrated HCl in mixed toluene and benzene solution resulted in C_60_H_36_ [57]. Furthermore, electrophobic addition was more likely in the case of carbon atoms bound with hydrogen. In particular, the oxidation of hydrogenated fullerenes was achieved by dissolution in toluene over a 48-hour period under ambient light [57]. In addition, the oxidation of fullerenes also occurs under UV irradiation (254 nm) [58]. 

The organic modification of oxidized C_60_ is performed to achieve covalent linkages between epoxy and C_60_. The silanization of C_60_ with 3-aminopropyltriethoxysilane was found to enhance molecular relaxation and the thermal behavior of the epoxy system [59]. A twofold enhancement was achieved in the mechanical properties of thermoplastic polyurethane filled with salinized C_60_ at low concentrations. 

Carboxyl, ester, and piperazine have also been attached to the C_60_ molecule through multi-step synthesis methods [60]. The improved solubilization of carboxylic acid fullerenes was achieved by their surface modification using titanium dioxide, via esterification (P_25_/C_60_-COOH or C_70_-COOH) technique [61]. Fischer esterification was also demonstrated as a possible route for the improved synthesis of fullerene derivatives [62]. Three derivatives that were functionalized with the 5, 7, and 9 morpholine groups were synthesized by the amination of Gd@C82 with morpholine [63]. 

Sulfurizations of fullerenes have been investigated using pure sulfur, thioacrylamides, thiadiazolidinones, benzothiet, dendrimer disulfide, dithianediol, and aryl isothiocyanates. The thiolation of fullerenes is rarely studied [64,65]. Thiolation with thiols is typically achieved via a reaction with multiple chlorinated fullerenes [66,67]. Recently, the thiolation of C_60_ with thiols conjugated to polystyrene was reported [68]. 

Multiple additions of an even number of fluorine atoms occur during the fluorination of fullerenes, resulting in a range of derivatives, from C_60_F_2_ to C_60_F_60_ [69]. Various fluorination reactions—from the extremely aggressive KrF_2_ to very mild fluorides—have been performed. The fluorination of C_60_ with cesium−lead oxyfluorides at 550°C resulted in a C_2_ loss and the formation of C_58_F_18_ and C_58_F_17_ [70]. 

Chlorofullerenes C_60_Cl_n_ (*n* = 8, 10, 12, 14, 24, 26, 30) have been synthesized and widely investigated [71,72]. The chlorination of C_60_ or C_70_ using an ICl solution produces the isomers of C_60_Cl_6_ and C_70_Cl_10_, respectively [73,74,75]. High-temperature chlorination of C_60_ results in the quantitative formation of C_60_Cl_24_, C_60_Cl_28,_ or C_60_Cl_30_. However, not all of the synthesized chlorofullerenes are stable in the air and can exist only under certain conditions. It was also reported that chlorofullerenes can be converted into C_60_F_l8_, C_60_F_l5_Ph_3_, C_60_Br_24_, C_60_F_24,_ and C_60_Cl_24_, and into many different products, including C_60_Me_6_ and C_60_Me_5_Cl, C_60_(OEt)_5_OH, and C_60_Ar_5_C_l8_ (Ar = Ph, tolyl, anisyl, tert-butyphenyl, thienyl, and fluorifenil).

The direct bromination of fullerene yielded either C_60_Br_8_, C_60_Br_14_, or C_60_Br_24_ [76]. The bromination of fullerenes in organic solvents yielded C_60_Br_6_ and C_60_Br_8_ [77]. Bromofullerenes are notoriously insoluble. The bromination of fullerene results in yellow-orange microcrystalline bromo-derivatives of the composition, C_60_Br_24_. Bromo-fullerenes with a lower atomic fraction of bromine, i.e., hexa and octa bromofullerenes, were also reported [77]. 

The functionalization of fullerenes with bioactive materials that exhibit an affinity to certain nucleic acids, proteins, or cell receptors, such as peptides or saccharides, functionalization with polar or ionic groups, such as hydroxy, carbonyl, or quaternary ammonium salts, and encapsulation with macromolecules are all highly suitable for various biomedical applications [78,79,80,81,82]. A fullerene-DNA conjugate was found to bind to the target DNA and might be useful for delivering DNA into living cells [81]. 

It was also reported that C_60_ may be used for lithium batteries as they showed the electrochemical intercalation of lithium into solid C_60_, which corresponds to the C_60_Li_12_ form. It can be effectively used for anode materials in lithium–ion batteries [83]. 

Finally, endohedral functionalization has attracted attention due to the unique properties it confers. After the discovery of endohedral fullerenes, such as Sc_3_N@Ih-C_80_, many other nitride cluster fullerenes have been reported, including scandium, yttrium, and most of the lanthanides [13]. The discovery of Gd_3_N@C_2n_ (*n* = 39–43) resulted in the synthesis of a wide range of members of this family of endohedral fullerenes [84,85,86]. Since the discovery of Sc_2_S@C_2n_ (*n* = 35–50), this family of fullerenes has attracted great scientific attention. In addition, a limited number of reports have been published about Ti_2_C_2_@D_3h_-C_78_ and Ti_2_@C_84_ [84,87]. Chen and co-workers isolated and characterized the metal carbide endohedral fullerene, Sc_2_C_2_@C_88_ [87,88].

## 4. Applications in Electrochemistry

The application of cyclic voltammetry in revealing the redox processes of fullerenes is the most commonly used application of electrochemistry. It provides information about the energy and electron transfer processes and their chemical reactions. It was shown that neutral C_60_ is capable of accepting up to six electrons via reduction [13,86,89]. Soon after the discovery of fullerenes, the cyclic voltammetry of C_60_ was reported [55]. Importantly, among all the carbon-based materials, only fullerenes are expected to be potential candidates for single-molecule electronic devices. 

The larger fullerenes have attracted attention due to the rich chemistry provided by the high symmetry and different distortion mechanisms. There is a systematic shift toward positive potential in cathodic electrochemistry from C_60_ to C_84_ fullerenes [90]. 

The surface modification of fullerene cages with various functional groups can lead to structural distortion, resulting in changed cyclic voltammograms. For example, the reduction potential of a multihydrofullerene (e.g., C_60_H_x_) is achieved because the embedded hydrogen atoms are more negative than those of pristine fullerenes.

The encapsulation of fullerenes with La, Gd, Tm, Y, Yb, Ce, Pr, and Sm results in unique electrochemical properties [91,92,93]. These electrochemical properties change due to charge transfer between metals and fullerenes. Many lanthanides can give three electrons to fullerenes, resulting in trivalent metallofullerenes. In addition, it was found that they can be more easily oxidized or reduced than empty fullerene cages. Linear dependence between the redox potential and ionic radii of the encapsulated metals was clearly observed. Interesting behavior was observed upon encapsulation with metals of different sizes. Smaller metals resulted in the formation of fullerenes that can be reduced and oxidized, in comparison to larger atoms, which were more difficult to reduce. With the increase in the size of fullerenes, the number of possible oxidation and reduction routes also increased. 

**Figure 6:**
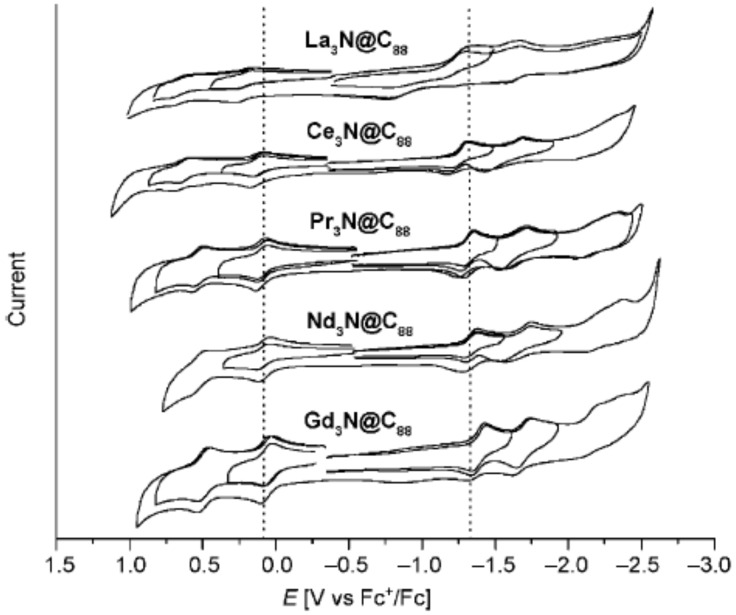
Cyclic voltammograms of M_3_N@C_88_(M = La, Ce, Pr, Nd, and Gd), with ferrocene as the internal standard.

Alkaline earth metals and some lanthanides showed the transfer of two electrons to fullerenes. The electrochemical gap for these fullerenes is larger than that of previously described fullerene structures [94,95]. Metal nitride-encapsulated fullerenes exhibit very similar electron-accepting properties that are independent of the metal that is encapsulated (Figure 6). The fullerenes encapsulated with Ce, La, and Er accepted six elections from the encaged atoms [96,97,98], showing relatively low electrochemical bandgaps. 

The large electrochemical bandgap is of fullerenes filled with nitride-based complexes [99]. In combination with lanthanides, such as Sm, Eu, Yb, Sc, and Y, the most abundant fullerenes have been achieved. It is important to note that some of these molecules, for example, Sc_3_N, are not stable under ambient conditions. However, being encapsulated in C_80,_ they show remarkable stability, accompanied by high yield and purity. This was proved by the moisture-resistive and highly electrically conductive performance of a constructed solar cell. In particular, the solar cell device showed a survival efficiency of over 17% after continuous illumination for 800 h. The electrochemical irreversibility observed for large metals, such as Nd, Pr, and Ce, suggests that structural reorganization may occur. 

Interestingly, only a few components showed a significant change during the oxidation and reduction steps depending on the nature of metal clusters encapsulated inside fullerenes [100,101]. The valence state of the Ti atoms in TiM_2_N@C_80_ resulted in changes in both processes, while CeM_2_N@C_80_ only affected oxidation. Therefore, the redox potential highly depends on the size of the metallic nitride complex. It was also shown that the oxidation process depends on the size and symmetry of the fullerene in which it was encapsulated. 

Metal carbides have become an important filling for fullerenes. The reduction or oxidation of Sc_3_C_2_@C_80_ alters only the charge of the C_2_ moiety, while the Sc and cage retain their respective charges of 3+ and 6. In addition, Sc_2_O@C_82_ and the sulfide Sc_2_S@C_82_ showed +4 charges on their carbon cage [102,103]. In these structures, the HOMOs are mostly localized on the carbon cage. This results in values close to their oxidation potentials.

Li@C_60_ is paramagnetic, with a small gap of 0.47 eV [104]. It has a stable form of Li^+^@C_60_. Li^+^@C_60_ has a cathodic shift of all reduction steps of 0.6 V, showing a similar reduction to pure pristine C_60_. It is easily oxidized and reduced, due to the unpaired electron on the fullerene cage. The fullerenes filled with Ca, Sm, Eu, Tm, and Yb are diamagnetic [105,106,107,108]. Their electrochemical gap reaches 1 V and they are electron acceptors. 

Interestingly, nitrogen-functionalized fullerenes filled with Y, Tb, and Gd showed paramagnetic properties [109]. An electrochemical study of Gd_2_@C_79_N demonstrated that it has two reduction potentials at -0.96 V and at -1.98 V. Using this observation and the DFT calculations, it was assigned to endohedral reduction. The oxidation potential of Gd_2_@C_79_N is similar to that of La_2_@C_80_.

Nitride-cluster fullerenes, such as Sc_3_N@C_80_, showed significantly different behavior in comparison to the nitride-filled fullerenes mentioned above. In comparison to Y_3_N@C_80_, they showed similar anodic and different cathodic behavior. They have similar oxidation potentials but different reduction potentials [110,111]. These two fullerenes have different reversibility in terms of the oxidation and reduction processes. Sc_3_N@C_80_ has the highest oxidation potential among the nitride-cluster fullerenes. The separation of two different isomers, I_h_ and D_5h_, was used to compare their electrochemical properties. It was shown that they have significantly different oxidation potentials [110]. 

Sc_3_N@C_80_ has also been widely investigated. In particular, it was shown that it can be further functionalized with CF_3_ and benzyl. These functional additions significantly affect the redox potential [112]. Interestingly, multiple CF_3_ additions shift the first reduction potential further in the positive direction, whereas further additions result in reversing the effect, due to the saturation of the π-system [113]. Ce-filled fullerenes showed significantly different behavior. It was found that Ce^III^-functionalized fullerenes are electrochemically inert [114]. 

Titanium and vanadium clusters inside fullerenes, such as TiSc_2_N@C_80_, TiY_2_N@C_80_, VSc_2_N@C_80_, and V_2_ScN@C_80_, showed similar reduction and oxidation. However, both processes are electrochemically reversible. For many fillings with a sixfold charged structure, C_80_ is the most abundant. Most of them showed low oxidation potential with a paramagnetic nature. As is similar for all sixfold clusters, reductions are usually electrochemically reversible. Sc_4_O_2_@C_80_ also exhibits reversible reduction and oxidation at 0.00 and −1.10 V [115]. 

Ti-based carbides with lanthanides are similar to nitrate complexes that are encapsulated inside fullerenes [115,116,117,118,119]. The oxidation potential of these fullerenes is close to 0.6 V. The reduction potential varies with the lanthanides’ ionic radius, in a predictable manner. The most positive reduction potential was found for Gd, while the most negative reduction potential was found for Sc. Knowledge of electrochemical properties is required in numerous applications. Significant attention was paid to the study of the electrochemical properties of fullerenes. 

For applications in electrochemistry, it is important to develop low-cost methods for the synthesis of fullerene derivatives. The most important property of these materials is capacity. High capacity will lead to excellent and, therefore, improved electrochemical characteristics. The biological compatibility present in a group of fullerene derivatives is important for electrochemistry, due to their potential for use in biological media. Another parameter that is important for use in biological and other systems is recyclability, which is high for fullerenes.

## 5. Application of Fullerenes in Solar Cells

The increasing demand for energy is a strong motivator for scientists to look for alternative and renewable sources of energy. The most obvious energy source, which can be converted into various applications, is radiation from the sun [120,121]. By virtue of the photovoltaic effect in semiconductors, it can drive devices for the direct conversion of light energy into electricity, via so-called solar cells. This effect is based on the photogeneration of charge carriers, which takes place when the light energy quantum is bigger than the bandgap in the absorbing semiconducting medium, which is usually a p-n junction. 

**Figure 7:**
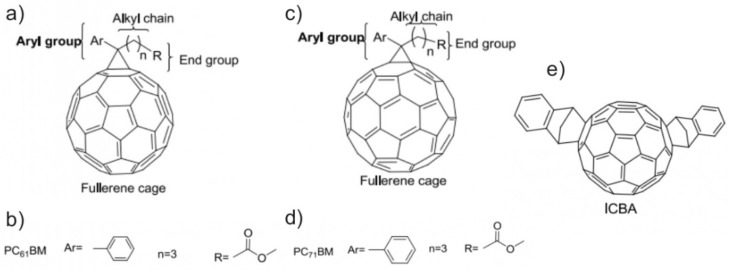
(**a**,**c**) Fullerenes C_60_ and C_70_ and their marked parts, which can be modified; (**b**,**d**) the fullerene derivatives PC61BM and PC71BM (see (**a**,**c**) for Ar, n, and R); (**e**) indene-based C60 bis-adduct [131]. Reprinted with permission of Elsevier (Copyright 2017).

Fullerenes (see Figure 7a,c) are known to be efficient electron acceptors, which makes them promising candidates for the construction of a solar panel when combined with P3HT as the donor of electrons [122,123,124,125]. Indeed, this material gives hope for high $V_{oc}$ and $I_{sc}$, for open and short circuits respectively. However, by themselves, fullerenes have low solubility and low-lying lowest unoccupied molecular orbital (LUMO) levels. Hence, their utility in high-performance solar cells hinges on chemical engineering. The improvement of the power conversion efficiency (PCE) of the organic solar cells (OSC) can be achieved via fullerene tailoring, which leads to broad and strong absorption, better solubility, and miscibility, while a high-lying LUMO level increases the $V_{oc}$ and gives a smooth morphology. The latter allows charge carriers to reach high mobility and obtain the significant, fill factor $\left(FF\right)$ of a solar cell [125]. 

PC_61_BM (see Figure 7b) might also be chosen as an acceptor [125,126]. Despite its low-lying LUMO levels, which are simply designed, it is easily prepared, soluble, and stable. The efficiency of a solar cell that is based on polymer-PC_61_BM BHJ can be enhanced by various chemical procedures, such as replacing the phenyl groups, varying the alkyl chain lengths, modifying the terminal ester group, and replacing the fullerene cage with highly absorbent fullerenes.

The first modification mentioned increases the thickness of the active layer, encouraging the elevation of FF, and offers better solution processing because of improved solubility. The rather high values of the short-circuit current in the OSC with modified PC_61_BM mono-adduct derivatives result in lower PCE. This finding was shown in a series of papers [127,128,129]. The alkyl chain length is related to the optical and electronic properties of the material, which affect the efficiency of a solar cell. It was shown that a reduction in the alkyl chain length influences the solubility and, consequently, lowers PCE, whereas other modifications induce high absorption and improve PCE (from 0.4 to 2.8%) [130,131,132]. End-group modification generally influences the absorbing properties of the acceptor, leading to better PCE values. Despite this fact, the mobility of charge carriers and the short-circuit current declined due to the rough morphology [133,134]. Finally, it is concluded that the modification of PC_61_BM with mono-adducts donates nothing specifically related to PCE, only slightly altering it. The same is applied to another fullerene derivative, PC_71_BM (see Figure 7c,d), due to the imperfections caused by the aforementioned adjustments [125,135,136,137]. 

In the case of multi-adducts, it was observed that the most fruitful results were achieved with bis-adduct derivatives. They showed high $V_{oc},$ explained by heightened LUMO levels and, hence, increased PCE compared to the pristine C_60_, modified with mono-adducts PC_61_BM [137,138,139,140,141]. On the contrary, tri-adducts and those of higher orders are expected to have many isomeric forms, which lessens $I_{sc}$ and FF and leads to small PCE. When PC_61_BM was replaced with PC_71_BM, a decline in all the parameters responsible for the efficiency of a solar cell was observed, due to shallow electron-trapping behavior. 

The aforementioned adducts are limited to PCE values of not more than 5%. The next generation of fullerene derivatives was slightly improved in this department. It has been shown recently that indene-C_60_ bis-adduct (see Figure 7e), which has a higher LUMO level than PC_60_BM and PC_70_BM, enhanced the PCE values up to 6.5% [125,142,143]. The problem of further PCE improvement lies in insufficient absorption in the required spectral range.

Although fullerene derivatives have been proven to be good acceptors, OSC still do not show high results for PCE, compared to inorganic ones. Conversely, solar cells based on perovskites are widely known for their high efficiency (over 22%), which is provided by their good donor properties [144,145]. These can be explained by the crystal structure, high charge carrier mobility, and long diffusion length, which supports strong light absorption and, hence, the production of a photocurrent. However, this material is not stable enough and demonstrates hysteretic behavior in the I-V curve. The nature of this effect lies in the time-dependent loss of output, which might be interpreted differently. 

**Figure 8:**
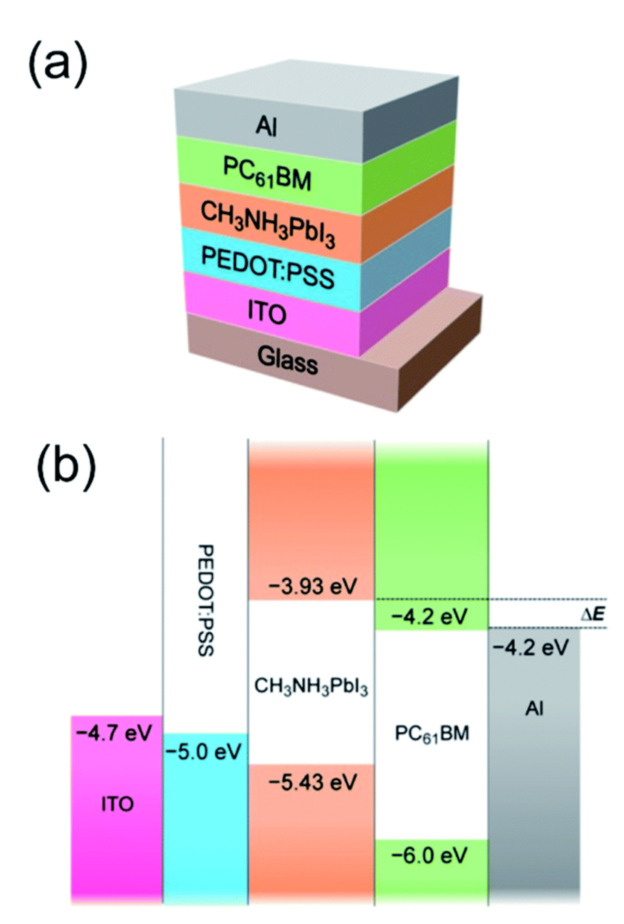
(**a**) Scheme of an inverted perovskite-fullerene-based solar cell; (**b**) energy diagram of the layers in the structure [146].

**Figure 9:**
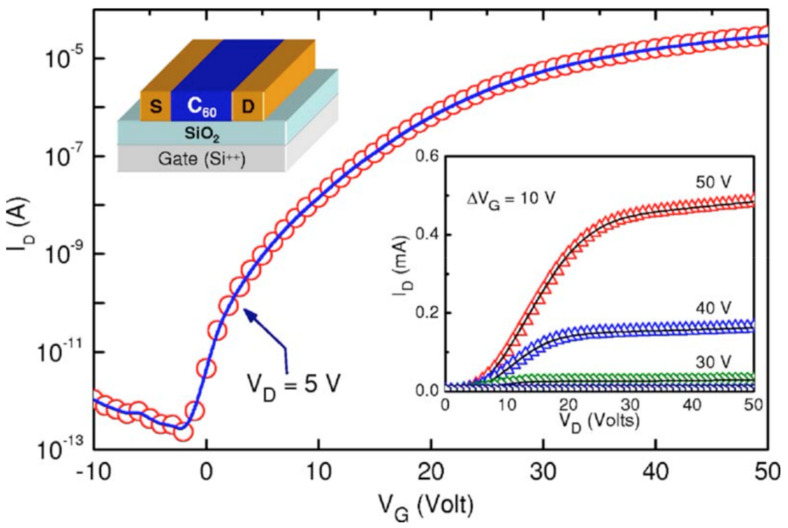
The typical curve of the source-drain current, versus source-gate voltage at a given source-drain voltage. Reprinted from [147], with the permission of AIP Publishing.

Recently, good donors and acceptors have been used together in so-called hybrid solar cells, which were found to improve the operation of the device. The typical scheme of the latter is the same as that shown in Figure 8a, where the polymer P3HT is changed to perovskite (mostly CH3NH3PbX3, which provides highly efficient light registration ability) (see Figure 9). It was discovered that the hysteresis in such a device became negligible and PCE reached 17.6% when PCBM played the role of an acceptor. When the fullerene bis-adduct, BAFB, was used instead, a PCE of 18.1% was achieved because of the completed quenching of perovskite photoluminescence. In addition, devices with PCBM and its modifications demonstrated rapid electron transport, and there was a perfect match between the LUMO level of PCBM and the edge of the perovskite conduction band, which implies good photovoltage output. 

The integration of fullerene as an electron transport layer can be difficult, due to several unsolved problems. Among them are the dissolution and thermal vaporization of this layer in the course of traditional manufacturing protocols. However, the creation of chemically functionalized fullerenes opens up the possibility of overcoming this problem. The selective interaction of functionalized fullerenes with perovskite surfaces has been widely investigated. A dimethylaminopropylamine-C60 multi-adduct lowered the work function of Ag, Cu, and Au and can be used in cathode buffer layers for inverted polymer solar cells that can operate in ambient conditions. In addition to the doping effect observed with the insertion of this layer, it also results in improved roughness. 

Another important role of functionalized fullerenes in solar cells is in avoiding issues due to the deposition of further layers on top of them. Some fullerene derivatives have already been used as passivating agents. For example, di-tert-butyl moieties create protective groups for two carboxylic acids, which ensure good solubility of the compound in organic solvents, enabling efficient solution-processing and the formation of homogeneous films on top of TiO_2_. After heating at 170 °C, these groups are eliminated, as the 2-methylpropene and carboxylic acids bind to the titania. This makes the coverage of fullerenes homogeneous on the top of the titania, in addition to its additional hydrophobicity. 

The wettability issue has been widely discussed in a number of research studies. The use of a dual modification of the TiO_2_ surface was proposed, employing two different C60 derivatives. The 6,6-phenyl-C61-butyric acid methyl ester, in combination with further ethanolamine-functionalized fullerenes, results in the passivation of the traps, with improved wettability properties. Functionalized fullerenes can then be used to protect from the solvents. Styryl units have been employed widely for this purpose. 

Thermal protection by using functionalized derivatives has also been reported. The derivative containing benzocyclobutene functionality, the phenyl-C61-butyric acid bezocyclobutene ester, was presented as a possible candidate in an n-i-p configuration similar to PSC, to be deposited directly onto the transparent conductive oxide, omitting the TiO_2_ layer, thereby allowing annealing, even at 200 °C. 

Scientific attention was attracted by the prospect of solving the issue with improved long-term stability. A functionalization strategy can be used to overcome this problem. Silane molecules bearing highly hydrophobic, fluorinated tails can self-assemble through hydrogen bonding interactions onto a pre-assembled C_60_. A solar cell with such a layer demonstrated significantly higher stability under environmental conditions. The new dimeric species proved to be able to effectively passivate surface traps and to extract electrons. The stability test showed a drop of 25% over 350 h for a dimer-functionalized fullerene device. 

A study on the effect of a series of novel fullerene derivatives with pronounced electron-accepting capabilities in terms of efficiency and stability has been performed. In particular, higher short-circuit current values were obtained in devices containing the novel strong electron-accepting fullerene derivatives. Isooxazoline [60] fullerenes have proven to be a good candidate for processing blend films with CH3NH3PbI3 and obtaining enhanced power conversion efficiency in polymer solar cells. Pyrazoline and methanol [60] fullerene derivatives versus pristine [60] fullerene results in improved performance. Furthermore, a clear correlation between the LUMO energy level of the fullerene component and the open-circuit voltage of the solar cells has been found.

## 6. Applications in Nanoelectronics 

In 1995 [148], it was shown that thin films of fullerene on the $\mathrm{Si}/{\mathrm{SiO}}_{2}$ substrate, with gold contacts on the top, provide n-type conductivity, with electron mobility estimated to be as low as $0.08\;\frac{{\mathrm{cm}}^{2}}{V\;s}$. In further works [149,150], organic thin-film transistor (OTFT) fullerene devices with bottom-contact geometry appeared to demonstrate electron mobilities of more than $2\;\frac{{\mathrm{cm}}^{2}}{V\;s}$. The dependence of the source-drain current versus source-gate voltage for fullerene OTFT is shown in Figure 9. 

Almost all of the reported results of electrical measurements on fullerene OTFTs were conducted in a high vacuum or nitrogen atmosphere. After air exposure, the device’s performance degrades dramatically. To stabilize the performance of the device, passivation with an insulating alumina layer. Nevertheless, the passivation also lowered the electron mobility in the device to 0.1 cm^2^/(V s), which is not quite good enough for practical applications. 

Fullerenes in their pure form have relatively low solubility in most widely used solvents. Hence, the device fabrication usually proceeds through vapor deposition onto the substrate. However, by using proper functionalization, it is possible to dissolve fullerene and use spin-coating to laminate it into a thin film. This was achieved using methanofullerene [6,6]-phenyl C61-butyric acid methyl ester. The resulting electron mobility was equal to 2–4 × 10^−3^ cm^2^/(V s), and this was improved to a value of 0.1–0.2 cm^2^/(V s).

Excellent conductivity properties, charge transport, and charge separation make fullerene an ideal material for sensors. It has been used in various sensors, such as strain and gas sensors, photoelectrochemical sensors, and electrochemical and optical sensors. In the demand for high performance from stretching devices, it is important to achieve highly linear and reversible ductability at any strain in the range (up to >50%). Such a high sensitivity has been developed in mixed-dimensional composites consisting of AgNWs, graphene, and fullerenes. Each of the components yielded some unique properties, such as high electric conductance, high strain sensitivity, and lubrication. The reversible ductile strain was achieved due to the presence of fullerenes, which provided slippage. A new sensor based on CNT, graphene, and fullerene has recently been developed. These allotropes of carbon result in high elasticity, accompanied by high conductivity. Berdinsky et al. have reported on fullerene-based sensors for temperature and pressure detection. The high-sensitivity response of the sensor to changes in temperature, humidity, and pressure has been recorded. The C_60_ shows photo activity when illuminated with light at a quantum energy close to 1.4 eV. Fullerene derivatives can be used in light-sensing devices for applications that range from motion control to medical imaging. 

The cage seen in fullerene derivatives has been used to trap small gas molecules. SnO_2_ quantum dots-C_60_ nanohybrids were used for the detection of gases, such as 1% of methane and propane and 70 ppm of H2S gas, over a wide temperature range. A ZnO/C_60_-based nano-composite device allows the detection of certain gaseous organics. such as ethanol. A microbalance humidity sensor, based on C_60_ and graphene oxide. 

Due to its excellent electrochemical sensitivity, C_60_ can be used for the detection of numerous biomolecules, in particular, dopamine, glucose, and uric acid. Piezoelectric-based immunosensors with C_60_ nanomaterials with very high sensitivity are able to detect IgG, reaching a detection limit of 0.0001 mg/mL. Another biosensor can be used to detect prostate-specific antigen (PSA) in a measuring range from 0.005 to 20 ng/mL. Impedimetric Fetuin biosensors modified with fullerene have also been developed. Using similar approaches, an impedimetric sensor system modified with cortisol-imprinted polymers on fullerene has been developed for the determination of cortisol in saliva. Low-amount measurement of miRNA-141 has been developed in another fullerene-modified biosensor study. The system’s ultra-high sensitivity was achieved with C_60_-modified gold electrodes that were modified with amino and thiol groups. 

The efficiency of different types of sensors can be tuned by fullerene functionalization with different materials. For example, carboxylated multi-walled carbon nanotube-conjured-C_60_ has been used for the immunosensing of carcinoembryonic antigens. Similarly, Au nanoparticles have been widely used for the improved sensing of antibodies, erythropoietin, with a low limit of detection. The surface of multifunctional reduced-graphene fullerenes was reformed with Pt@AuNPs and a thiolated SDM-binding aptamer, which led to the high selectivity of the sulfadimethoxine sensor, linear response, and a low detection limit of around 8.7 fg/mL. Fullerene doped with a poly(amidoamine) (PAMAM)-functionalized metal-organic framework has been used to form a new nanohybrid of C_60_@PAMAM-MOF, which exhibits remarkable redox activity and an improved electrochemical response signal. Lu et al. developed a sensor based on gold NPs @ C_60_ (AuNPs@C_60_) for sensing phenolics. 

## 7. Conclusions

Despite the fact that fullerenes were discovered in the 1980s, they still play an important role in applications in electrochemistry, solar cells, and nanoelectronics. From both a theoretical and an experimental viewpoint, it is important to expand our knowledge about the interaction of fullerenes with different functionalities. This will enable researchers to overcome the major roadblocks present in the application of fullerenes—modest solubility and the reactivity of fullerenes to moisture. Developing novel functionalization routes for fullerenes can improve device efficiency and stability, which is desirable but challenging, due to the difficulty in precise control in terms of the high-selectivity grafting of suitable functional groups and their addition patterns. It is important to note that fullerenes have highly adjustable electronic properties that can be tailored by varying their size and functionalization. This property can be used to construct novel fullerene derivatives with appropriate energy levels, which is important for numerous applications. Furthermore, the high solubility of fullerenes in solvents suggests that they might be used in large-area devices. 

## Data Availability

Not applicable.
